# Twin domain imaging in topological insulator Bi_2_Te_3_ and Bi_2_Se_3_ epitaxial thin films by scanning X-ray nanobeam microscopy and electron backscatter diffraction

**DOI:** 10.1107/S1600576717000565

**Published:** 2017-02-17

**Authors:** Dominik Kriegner, Petr Harcuba, Jozef Veselý, Andreas Lesnik, Guenther Bauer, Gunther Springholz, Václav Holý

**Affiliations:** a Faculty of Mathematics and Physics, Charles University, Ke Karlovu 5, 121 16 Prague 2, Czech Republic; b Institut für Experimentelle Physik, Otto-von-Guericke Universität Magdeburg, Universitätsplatz 2, 39106 Magdeburg, Germany; c Institute of Semiconductor and Solid State Physics, Johannes Kepler University Linz, Altenbergerstrasse 69, 4040 Linz, Austria

**Keywords:** twinning, electron backscatter diffraction, scanning X-ray diffraction, topological insulators

## Abstract

The twin distribution in topological insulators Bi_2_Te_3_ and Bi_2_Se_3_ was imaged by electron backscatter diffraction (EBSD) and scanning X-ray diffraction microscopy (SXRM). The crystal orientation at the surface, determined by EBSD, is correlated with the surface topography, which shows triangular pyramidal features with edges oriented in two different orientations rotated in the surface plane by 60°. The bulk crystal orientation is mapped out using SXRM by measuring the diffracted X-ray intensity of an asymmetric Bragg peak using a nano-focused X-ray beam scanned over the sample. By comparing bulk- and surface-sensitive measurements of the same area, buried twin domains not visible on the surface are identified. The lateral twin domain size is found to increase with the film thickness.

## Abstract 2

Imaging with surface- and bulk-sensitive electron and X-ray diffraction based microscopic techniques enabled identification of the twin domain distribution of Bi_2_Te_3_ and Bi_2_Se_3_ thin films. Correlations between the surface topography and crystal orientation are established.

## 1. Introduction   

**Figure 1:**
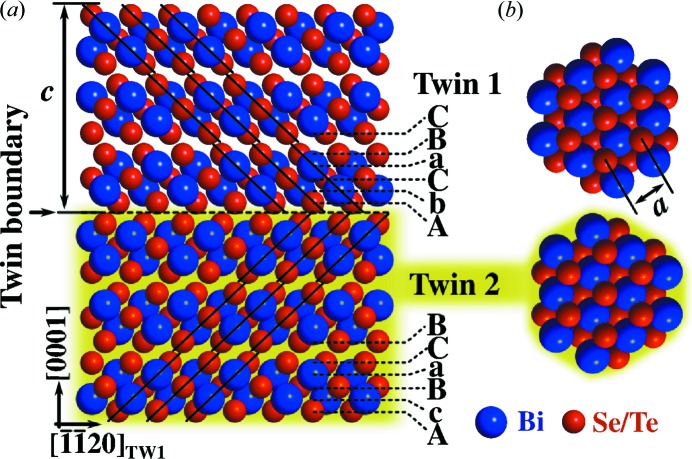
Sketch of the atomic structure of Bi_2_ *X*_3_ (*X* = Se, Te). Panel (*a*) shows the stacking of the basal planes for the two twin modifications. A unit cell consists of 15 atomic planes which are grouped into three quintuples. A dashed line marks a horizontal twin plane which separates twins 1 and 2 and marks the moment of a change of the stacking order, which is indicated. Panel (*b*) shows the top view of the two twin modifications. The unit-cell parameters *c* and *a* are indicated in panels (*a*) and (*b*), respectively.

Three-dimensional topological insulators represent a new state of matter with bulk band gap and Dirac cone-like surface states  (Zhang *et al.*, 2009 ▸). Bi_2_ *X*_3_ with *X* = Se and Te are prime members of this material class and were shown to exhibit the predicted exotic topological properties  (Chen *et al.*, 2009 ▸). For electrical devices, large-area high-quality thin films are required. However, epitaxial films as well as bulk crystals generally suffer from a large number of structural defects. The most common defects are twin boundaries that arise from the rhombohedral structure of Bi_2_ *X*_3_, which consists of three *X*–Bi–*X*–Bi–*X* quintuplet layers separated by a van der Waals gap. The stacking of the (000.1)^1^ basal planes can be described by a face-centred cubic (f.c.c.)-like *AbCaBC*… stacking as shown in Fig. 1 ▸(*a*), where lower- and upper-case letters correspond to Bi and *X* atomic layers, respectively. Because of the weakly bonded van der Waals gap, the Bi chalcogenides are highly prone to twin formation; a twinned domain (TD) exhibits the inverted *AcBaCB*… stacking and can be described as being rotated by 60° around the [000.1] *c* axis of the crystal with respect to that of the ordinary domain (OD). The boundary dividing the OD and TD, *i.e.* the twin boundary, is the most common planar defect in these materials and it can be parallel or inclined to the sample surface. Twin boundaries perpendicular to the *c* axis, which is the usual growth direction, are particularly common since the change of the stacking can easily occur in the weakly bound van der Waals gap formed by the *X*–*X* double layers, as shown in Fig. 1 ▸(*a*). However, in thin films twin boundaries inclined to the *c* axis also occur owing to the simultaneous nucleation of two different domains on lattice type mismatched substrate materials  (Schreyeck *et al.*, 2013 ▸; Tarakina *et al.*, 2014 ▸). Since it is the (000.1) surface of Bi_2_ *X*_3_ that features the topological surface states, the impact of such defects depends on their orientation. For example, (000.1) twin planes parallel to the surface were shown to influence the electronic properties of the surface state when they are in close vicinity to the surface  (Aramberri *et al.*, 2015 ▸). On the other hand, little is known about the impact of the lateral twins with inclined twin boundaries which cross the surface and therefore might perturb the surface state as well.

Up to now, only a few studies have dealt with the real structure and defects in epitaxial Bi_2_ *X*_3_ films, which will obviously depend strongly on the substrate material and growth conditions. Richardella *et al.* (2015 ▸) studied crystal defects in epitaxial layers of (Bi,Sb)_2_(Te,Se)_3_ on InP(111)B and SrTiO_3_ substrates using atomic force microscopy (AFM), transmission electron microscopy (TEM) and X-ray diffraction (XRD). These authors detected the presence of twin domains and interpreted the broadening of the XRD maxima as lattice mosaicity; they concluded that lattice mismatch between the substrate and the layer is the main factor determining the quality of the epitaxial growth and consequently the presence of twin domains. However, no information on the domain size and their arrangement was obtained. Reduced twin formation in Bi_2_Se_3_ was reported by Tarakina *et al.* (2014 ▸) by deposition on artificially roughened InP(111)B substrates. Another strategy for the twin suppression was chosen by Kampmeier *et al.* (2015 ▸), where Bi_2_Te_3_ layers were deposited by van der Waals epitaxy on Te-passivated Si(111) substrates using an extremely slow growth rate of 2.7 nm h^−1^. The deposited layer followed the threefold symmetry of the substrate surface and no twin domains were detected. For vicinal InP(111)A substrates Guo *et al.* (2013 ▸) have found that the miscut steps play a stabilizing role in the step-flow growth mode which suppresses the twin domains as well. In the mentioned work the existence of twin domains was detected by XRD pole-figure measurements and microscopically observed by cross-sectional high-resolution scanning transmission electron microscopy. However, no information on the sizes of twin domains and their vertical and lateral arrangement in the layer could be derived.

The aim of this study is to address and clarify this issue by real-space imaging of individual twin domains by electron backscatter diffraction (EBSD), TEM and scanning X-ray nanobeam diffraction microscopy (SXRM). The latter is based on reciprocal-space mapping using a highly focused primary X-ray beam with a footprint of about 200 nm, which is laterally scanned over the sample, as has been previously used for investigation of various thin layers and nanostructures (see, for instance, Stangl *et al.*, 2009 ▸; Hrauda *et al.*, 2011 ▸; Chahine *et al.*, 2014 ▸, 2015 ▸; Schäfer *et al.*, 2016 ▸). Here, we show that EBSD and SXRM are able to directly determine the local crystalline orientation with high spatial resolution. With EBSD, however, only the material in close vicinity to the surface is probed. The EBSD measurements are therefore correlated with the surface topography, whereas SXRM yields information from the whole film thickness. Combining the results with cross-sectional TEM investigations we obtain a complete picture of the twin domain/defect structure of both topological insulator Bi_2_Se_3_ and Bi_2_Te_3_ films. The paper is structured as follows: after description of the sample growth and the methods employed, we present our results for Bi_2_Te_3_ films and describe the evolution of twinning in Bi_2_Se_3_ as a function of the layer thickness. Our results reveal for the first time the twin distribution in such films and demonstrate that EBSD and SXRM are key tools for characterization and optimization of van der Waals bonded topological crystalline insulator heterostructures.

## 2. Experimental   

### 2.1. Sample growth and characterization   

Thin film samples of Bi_2_ *X*_3_ (*X* = Se, Te) were grown using molecular beam epitaxy on cleaved BaF_2_(111) substrates from compound BiSe/BiTe and elemental Se and Te beam flux sources. By adjusting the Se and Te fluxes  (Caha *et al.*, 2013 ▸), the growth of the pure Bi_2_ *X*_3_ phase is achieved with lattice parameters of *a* = 0.414, *c* = 2.865 nm and *a* = 0.438, *c* = 3.050 nm for Bi_2_Se_3_ and Bi_2_Te_3_, respectively. The lattice mismatch between the (111) BaF_2_ surface and the (000.1) *c* planes of Bi_2_ *X*_3_ is −5.5% and <0.2% for *X* = Se and Te, respectively. All epilayers grow with their *c* axis parallel to the [111] surface normal of the substrate. The larger mismatch in the case of the Bi_2_Se_3_ on BaF_2_ is easily overcome because of the weak links of the van der Waals gaps in the material, which has been proven to enable growth of this material on a wide range of different substrate materials (Richardella *et al.*, 2010 ▸; Taskin *et al.*, 2012 ▸; Guo *et al.*, 2013 ▸; Schreyeck *et al.*, 2013 ▸; Tarakina *et al.*, 2014 ▸; Kampmeier *et al.*, 2015 ▸). We study two different Bi_2_Te_3_ (BT) samples grown at different substrate temperatures and three Bi_2_Se_3_ (BS) samples grown simultaneously up to different layer thicknesses as listed in Table 1 ▸. For the Bi_2_Te_3_ sample BT-A the substrate temperature was about 40 K lower than for BT-B.

**Figure 2:**
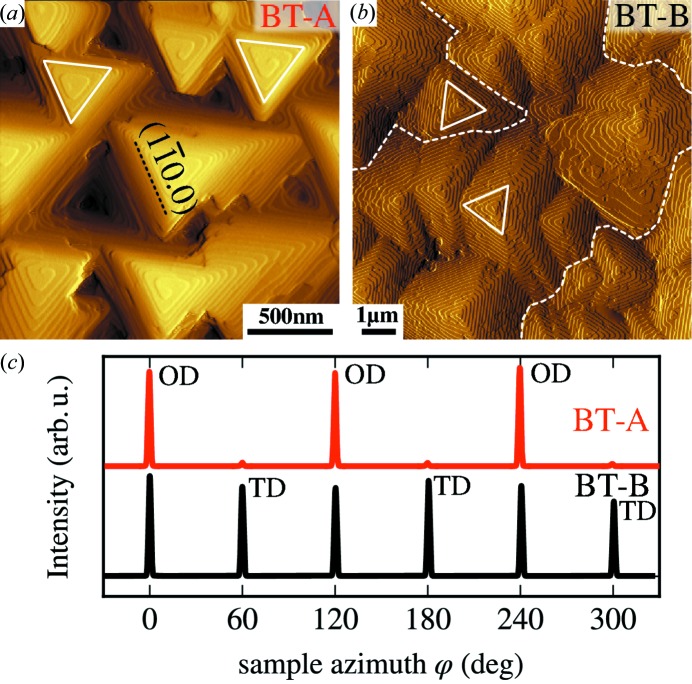
Surface characterization by AFM (*a*), (*b*) and large-area XRD azimuthal scans (*c*) of Bi_2_Te_3_ samples BT-A and BT-B. Both samples show triangular pyramids on the surface; however, for sample BT-B the pyramids show two orientations different by 60/180°. The lattice plane indices of the preferred surface step orientation are indicated in (*a*). White dashed lines in (*b*) mark the boundary between areas with the two distinct orientations. (*c*) XRD azimuth scans of the (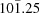) Bragg peak, which has a threefold symmetry according to the crystal structure, show that sample BT-A from panel (*a*) (red) indeed shows this threefold symmetry, while sample BT-B (black) shows a sixfold symmetry. For the X-ray measurements the signal from an area of ∼4 mm^2^ is averaged. The origin of the Bragg peaks from the ordinary domains and twin domains is indicated by OD and TD, respectively.

The surface morphologies of the films typically show a triangular pyramidal structure as exemplified by the AFM image of Bi_2_Te_3_ film BT-A in Fig. 2 ▸(*a*). The observed side steps of the pyramids are 1 nm high, which corresponds exactly to the thickness of one quintuplet (one-third of the crystallographic unit cell). These steps are oriented along 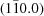 lattice planes as indicated in Fig. 2 ▸(*a*). Note that the 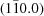 and 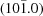 lattice planes differ because of the rhombohedral symmetry of the unit cell  (Nakajima, 1963 ▸). Depending on the growth conditions we find that the orientation of the triangles varies. For sample BT-A all visible pyramids are oriented in the same direction, whereas for sample BT-B (Fig. 2 ▸ *b*) two types of orientations are observed. This difference is caused by the different growth temperature and corresponds to drastically different XRD azimuth traces as shown in Fig. 2 ▸(*c*). These azimuthal scans were recorded at the (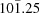) Bragg peak of Bi_2_Te_3_, which according to the rhombohedral crystal structure has a threefold symmetry. Indeed, a predominantly threefold symmetry is observed for sample BT-A, indicating an almost complete absence of any twinning, in perfect agreement with the presence of only one orientation of the surface pyramids in the AFM image. The majority of the diffraction signal of the (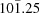) Bragg peak is found at 0, 120 and 240°, *i.e.* in the azimuth of the (224) substrate peaks, and corresponds to the ODs. Only around 3% of the total scattered X-ray intensity is found at azimuths of 60, 180 and 300°, and this stems from the TDs. In contrast, for sample BT-B an almost perfect sixfold symmetry of the (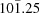) peak is found in the azimuthal scan, indicating a strong twinning in the thin film with almost equal abundance of the ordinary and twin regions. Note that the azimuthal scans were compiled from several scans performed at slightly different incidence angles to avoid inaccuracies arising from possible misalignments of the [000.1] direction from the goniometer rotation axis.

### 2.2. Twin imaging methods   

**Figure 3:**
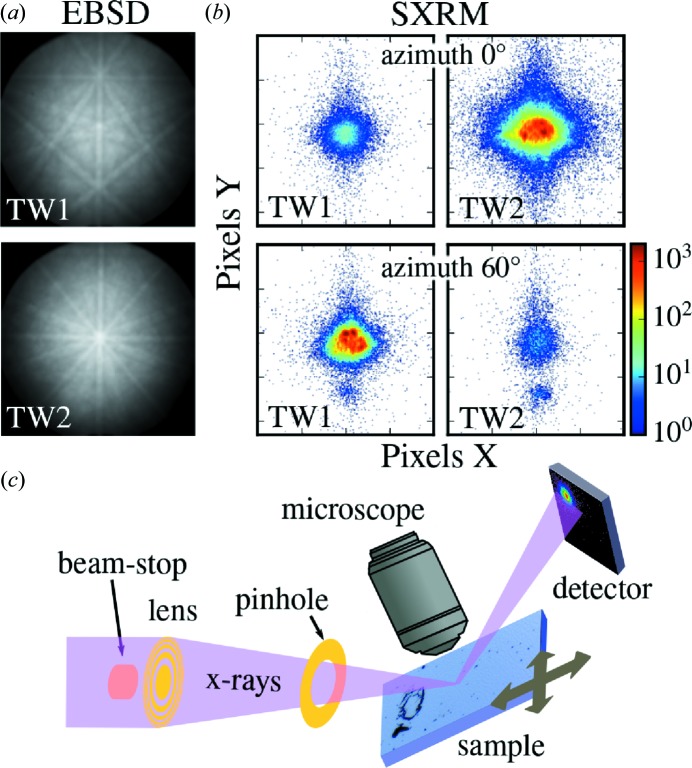
Twin contrast in EBSD and SXRM. (*a*) EBSD detects the two twin orientations by their two different Kikuchi patterns resulting from the different crystallographic orientations. The central pole corresponds either to the 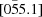 or to the 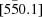 directions for twins 1 and 2, respectively. (*b*) In SXRM, performed at the asymmetric (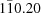) Bragg peak, different intensities are detected if the X-ray beam hits a domain of twin 1 or 2. Only one domain fulfils the Bragg condition in one azimuth as seen in the four (magnified) detector images. (*c*) Sketch of the SXRM setup at beamline ID01 at the ESRF in Grenoble, France, showing the focusing Fresnel lens, optical alignment microscope and two-dimensional X-ray detector.

To further investigate the correlation between surface morphology and twin orientation, microscopic methods directly sensitive to the crystal orientation are needed. For this purpose we used two complementary methods. First, we used the well established EBSD method, which, by recording the Kikuchi pattern from backscattered electron diffraction in a scanning electron microscope, is able to determine the local crystal orientation (Schwartz *et al.*, 2009 ▸). For EBSD measurements a Zeiss Auriga cross-beam electron microscope combined with a focused ion beam (FIB) was used. The microscope is equipped with a gas injection system for Pt layer deposition and an EDAX Digiview EBSD camera. Since the orientation of the two twins in our sample differs by a 60° rotation around the *c* axis, the two twin domains cause two distinctly different Kikuchi patterns as shown in Fig. 3 ▸(*a*). The TSL *OIM Analysis* software (http://www.edax.com/Products/EBSD/OIM-Data-Analysis-Microstructure-Analysis.aspx) used identifies the crystal orientation from these Kikuchi patterns using the crystal structure as an input. The information depth of this method is limited to the penetration/escape depth of quasi-elastically scattered electrons, which for the used operation voltage of up to 20 kV is limited to a few nanometres. Two different measurement geometries were employed to study the lateral and vertical twin distribution. For the lateral (plan-view) imaging, samples were studied using 20 keV electrons incident on the surface under an angle of 70°. For the cross-sectional investigations, samples were vertically cut using FIB milling after locally depositing a protective platinum layer. The side face of the cut was analysed by EBSD with the beam voltage reduced to 8 keV in order to minimize the electron beam induced charging effects and image distortions caused by the electron beam hitting the non-conductive substrate, and to increase the lateral resolution.

Hard X-ray photons of 8.5 keV were used as a probe for SXRM. In contrast to the low penetration depth of electrons, these penetrate the full film thickness. For SXRM the diffracted intensity of selected Bragg peaks was recorded from a very small spot of the sample using a tightly focused X-ray beam  (Stangl *et al.*, 2009 ▸). Focusing was achieved by a 1000 nm-thick tungsten Fresnel lens with a diameter of 300 µm and outermost zone width of 80 nm, which was fully illuminated and placed ∼10 cm upstream of the sample. This setup results in a ∼150 × 150 nm full width at half-maximum beam size at the sample position. Using a piezo-scanning stage and a two-dimensional MAXIPIX detector (516 × 516 pixels with a size of 55 µm mounted at a distance of 70 cm) the diffracted intensity can be recorded for various real-space sample positions (see Fig. 3 ▸ *c*), therefore creating an SXRM image with diffraction contrast. This can be repeated for different incidence angles of the primary beam to produce a five-dimensional data set consisting of three reciprocal-space dimensions 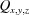 and two real-space dimensions 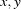. In our study the possible fifth dimension, *i.e.* the incidence angle, is only used to set the sensitivity to a certain Bragg peak. Fig. 3 ▸(*c*) shows a sketch of the SXRM setup at beamline ID01 at the European Synchrotron Radiation Facility (ESRF) in Grenoble, France, and for further details the reader is referred to the work of Chahine *et al.* (2014 ▸). To produce contrast between the two twin orientations the incident angle and detector position are adjusted to an asymmetric Bragg diffraction where either twin 1 (TD) or twin 2 (OD) diffracts. To align the sample to this condition, information on the crystal orientation and the lattice parameters is needed. For our epitaxial thin films these parameters were determined by laboratory XRD experiments which also provide the epitaxial relation to the substrate. When the focused X-ray beam hits the domain which diffracts only in a certain sample azimuth, a large intensity is found on the detector. In Fig. 3 ▸(*b*) this is the case for twin 2 for an azimuth of 0°, whereas for twin 1 the Bragg condition is not fulfilled. Rotating the sample by 60° (or 180°, or 300°) the situation is inverted as is shown in Fig. 3 ▸(*b*). Note that, owing to the lateral tails of the focused X-ray beam, a small diffracted intensity is always detected; however, the contrast between the two orientations is more than two orders of magnitude. Also note that, because of the divergence of the focused X-ray beam of ∼0.1°, resulting from the short focal length of the Fresnel zone plate, a small misalignment due to mosaicity does not influence the SXRM measurements.

## 3. Twin analysis of Bi_2_Te_3_   

### 3.1. Surface topography–twin orientation relation   

**Figure 4:**
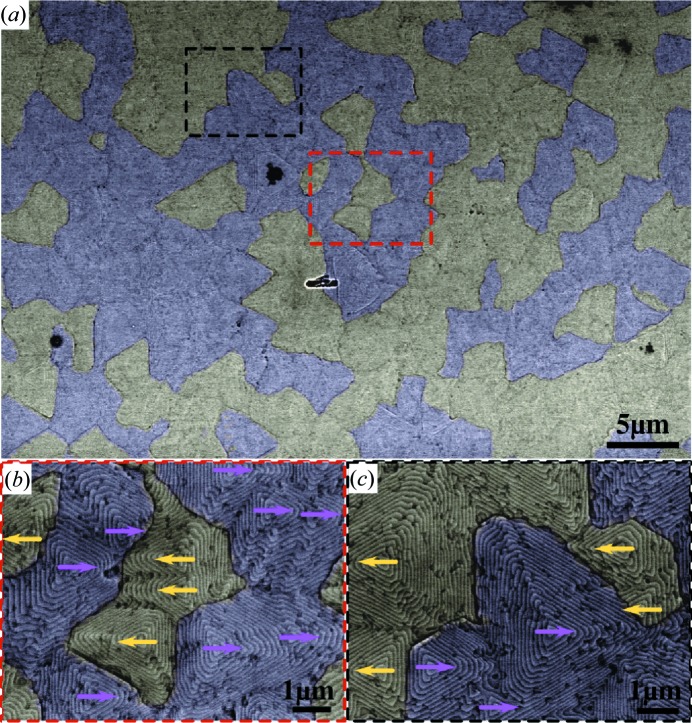
Surface morphology and twin domain correlation. (*a*) Scanning electron micrograph of sample BT-B overlaid with a semi-transparent representation of the EBSD crystal orientation data. The crystal orientation indicates the direction of the in-plane *a* direction/[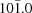] and in agreement with our XRD data finds only two types of orientations, *i.e.* the two twin orientations. The twin domain boundaries are correlated with the surface structure. Panels (*b*) and (*c*) whose location is indicated in panel (*a*) show enlarged details confirming this correlation. For clarity, arrows mark the orientation of the pyramids.

To correlate the surface morphology with the local crystalline orientation, we performed EBSD mapping and scanning electron microscopy (SEM) imaging of the same sample area to obtain the crystalline orientation map and surface topography. In Fig. 4 ▸(*a*) we show the SEM image of a ∼50 × 30 µm region in which the in-plane crystal orientation map determined by EBSD is overlaid as a semi-transparent coloured layer, where the violet and yellow colours correspond to the OD and TD, respectively. With EBSD we find only two distinct crystal orientations corresponding to the two 60°-rotated twin orientations. The borders of these areas are precisely correlated with the borders of areas showing one particular orientation of the surface pyramids as indicated by the triangles. This correlation can be better seen in the magnified areas shown in Figs. 4 ▸(*b*) and 4 ▸(*c*). All areas that are coloured yellow show the triangular pyramids pointing to the left, while areas that are overlaid by violet are pointing to the right. This proves that the surface topography is indeed correlated with the local crystal orientation of the thin film. From large-area EBSD measurements (20 000 µm^2^) the average size of the domains can be determined. For this purpose the average number of domain defects in every direction is determined and the resulting size is listed in Table 1 ▸. For sample BT-A such a determination is hindered by the strongly differing sizes of ODs and TDs. The ODs in sample BT-A cover most of the surface area as seen in Fig. 2 ▸(*a*). Since EBSD is only sensitive to the first few nanometres of the thin film, however, nothing can be concluded about the possible twin boundaries parallel to the surface or about the propagation of the defects into the depth of the thin films.

### 3.2. Scanning nanobeam X-ray diffraction microscopy   

**Figure 5:**
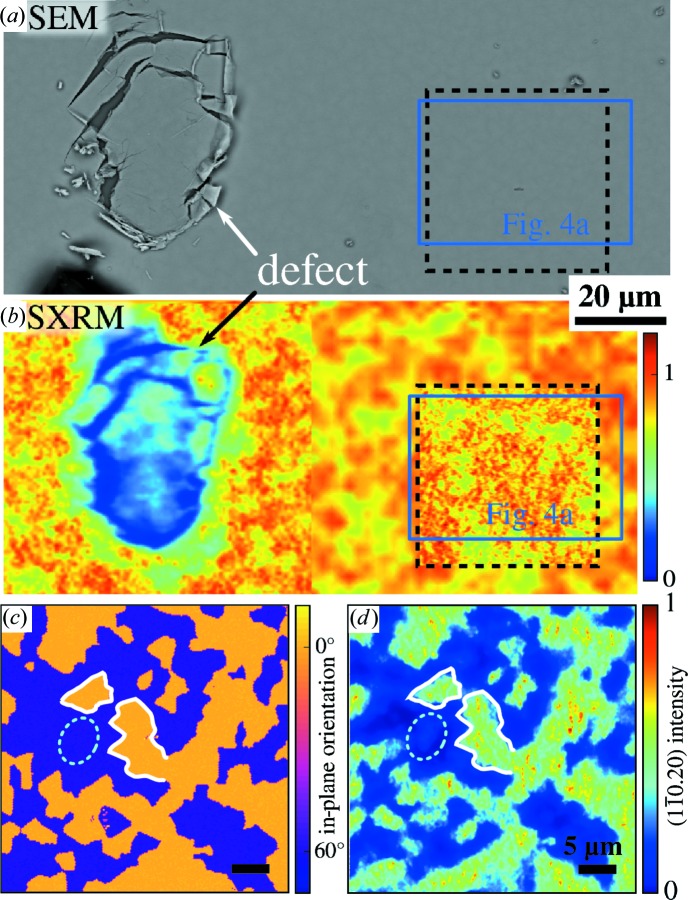
Comparison of SXRM and EBSD twin maps of the same position on Bi_2_Te_3_ sample BT-B. SEM (*a*) and SXRM (*b*) using a symmetric Bragg peak are used to localize the same area on the sample surface for both methods. The position of the EBSD map shown in Fig. 4 ▸(*a*) is marked by a blue rectangle, while a dashed rectangle indicates the location of the EBSD/SXRM measurements shown in (*c*) and (*d*), respectively. For the SXRM image in (*b*) the symmetric (000.15) Bragg peak was used to be insensitive to the twin domain pattern. (*c*) and (*d*) show the in-plane crystal orientation determined by EBSD, *i.e.* the direction of the *a* axis/[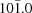], and an SXRM image of the same area produced by the asymmetric (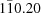) Bragg peak. The white lines in (*c*) and (*d*) mark the same features while the dashed ellipse highlights some distinctions.

Bulk-sensitive SXRM was employed to determine the crystalline orientation integrated along the X-ray beam trajectory, which penetrates through the full film thickness. Using an optical microscope for navigation and a macroscopically large defect (scratch) on the sample surface as a marker, we located the same position on the sample as already studied by EBSD. Figs. 5 ▸(*a*) and 5 ▸(*b*) show the corresponding SEM and SXRM images of this sample area, where the actual measurement areas of EBSD and SXRM are indicated by the blue and black dashed rectangles, respectively. Since inside the scratch the film material is either missing or tilted out of the Bragg condition, it shows up as a dark region in the SXRM image when the detector and incidence angle are set to the symmetric (000.15) Bragg reflection of Bi_2_Te_3_, which is insensitive to the twin orientation. The remaining areas in the (000.15) SXRM image show a rather homogeneous intensity distribution, indicating that all film parts are oriented with the *c* axis perpendicular to the surface. The remaining small intensity variations are most likely due to slightly varying film thickness since the surface pyramids observed by AFM in Fig. 2 ▸(*b*) typically have a height of several quintuple layers and therefore result in a film thickness variation of up to about 10%. The SXRM image in Fig. 5 ▸(*b*) was recorded with a step size of 2 µm, whereas the resolution limit given by the footprint of the X-ray nanobeam is roughly ∼400 × 150 nm at the (000.15) Bragg angle.

To investigate the twin distribution, the goniometer is set to the asymmetric (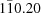) Bragg peak and SXRM images of the same area were recorded. At the asymmetric Bragg peak the lateral resolution of the SXRM is improved owing to the high-incidence co-planar measurement geometry resulting in a reduced beam footprint below 200 nm. Accordingly, in the SXRM images a step size of 160 nm was used for data acquisition. This is sufficient to study the micrometre-sized twin domain features seen in the EBSD map. Figs. 5 ▸(*c*) and 5 ▸(*d*) compare the EBSD map and the SXRM image of the same position for Bi_2_Te_3_ (BT-B), where the latter was recorded in the diffraction azimuth of twin 1 (0°). As indicated by the white lines, the EBSD and SXRM images are obviously highly correlated and the same features can be easily identified. However, distinct differences can also be seen. While at the surface, which is imaged by EBSD, one either finds only one or the other twin orientation, the SXRM intensity shows a more continuous variation. In particular, there are areas (an example is marked by a cyan ellipse) in which the diffracted intensity of twin 1 is observed in SXRM, while the EBSD image indicates the presence of the 60°-rotated twin 2 domain at the surface. This clearly indicates that the twin distribution across the film thickness differs from that at the surface. Note that the intensity in SXRM can vary continuously, whereas the nature of the EBSD data analysis imposes the limitation that only one particular crystal orientation can be determined for one measurement spot. If the contributions from two twin orientations are superimposed in the Kikuchi pattern the analysis software either neglects the weaker contribution or even fails to determine a distinct orientation for this particular measurement spot. Such an overlap of Kikuchi patterns was observed only near twin domain boundaries; however, in almost all cases the analysis software automatically identifies the stronger contribution. We highlight that within twin domains where SXRM detected a buried domain [*e.g.* as marked by the cyan ellipse in Figs. 5 ▸(*c*) and 5 ▸(*d*)] no such overlap of Kikuchi patterns was found.

**Figure 6:**
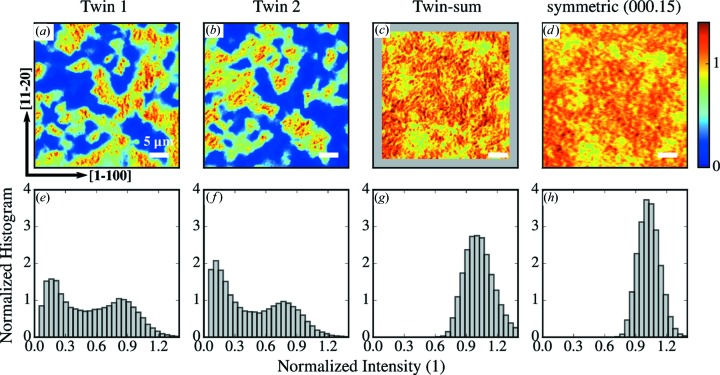
SXRM images of the same area of Bi_2_Te_3_ sample BT-B recorded with different Bragg reflections. (*a*), (*b*) Images of the normalized diffracted intensity of the (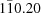) Bragg peak are shown for two azimuths different by 60°. The sum of the two images is shown in (*c*) and compared to the intensity obtained at the symmetric (000.15) Bragg peak in (*d*). Panels (*e*)–(*h*) show the corresponding intensity histograms. The normalization of the asymmetric peak intensities is performed in such a way that the histogram of the sum in (*g*) has a centre of mass of 1.

To clarify the origin of the SXRM intensity in locations where EBSD detects the other twin orientation, SXRM of the same sample area was also recorded in the 60° azimuth to detect a possible contribution of twin 2 at each measurement spot. Figs. 6 ▸(*a*) and 6 ▸(*b*) show the corresponding SXRM images using the asymmetric Bragg peaks of twins 1 and 2. One can see that at locations where a high signal is detected for twin 1 a low signal is found for twin 2, and *vice versa*. To confirm this complementarity, we sum both images in Fig. 6 ▸(*c*) and compare the result with the intensity of the symmetric (000.15) Bragg peak (Fig. 6 ▸ *d*) at which both twin orientations contribute equally. Both the sum of the asymmetric peaks recorded in two azimuths and the SXRM of the symmetric peak show a rather homogeneous distribution of the intensity with only weak recognisable features which are, however, very similar in both cases. Note that both images were recorded with the same step width, so the blurring in Fig. 6 ▸(*d*) as compared to Fig. 6 ▸(*c*) is a result of the change of incidence angle between the two SXRM measurements.

The SXRM images in Figs. 6 ▸(*a*)–6 ▸(*d*) are complemented by the intensity histograms shown in Figs. 6 ▸(*e*)–6 ▸(*h*). The histograms reveal that the intensity distribution of the asymmetric Bragg peak has contributions for all intensity intervals between 0 and the highest detected intensity. In the case when the twin domain boundaries always fully penetrate through the entire film thickness from the substrate interface to the surface, the film would either diffract or be out of the Bragg condition. Thus, a purely bimodal intensity distribution with normalized intensities close to either 0 or 1 would be formed. We find, however, more continuous-intensity histograms of both individual asymmetric SXRM intensity maps as shown in Figs. 6 ▸(*e*) and 6 ▸(*f*). As expected from the almost perfect sixfold symmetry of the azimuthal scan in Fig. 2 ▸ averaged over a very large film area, the histograms of the two azimuths are almost equal, reflecting the equal twin population in this sample. The histogram of the sum in Fig. 6 ▸(*g*), however, shows a well defined peak around 1 similar to the histogram of the symmetric Bragg peak (Fig. 6 ▸ *h*). From this we conclude that, in areas where for one twin the intensity is lowered, it is increased by the same amount for the other. This points to a more complicated vertical twin distribution in the bulk of the films as compared to the bimodal twin distribution at the surface and suggests that with surface-sensitive techniques not all twin domains are detected.

### 3.3. Cross-sectional electron backscatter diffraction twin imaging   

**Figure 7:**
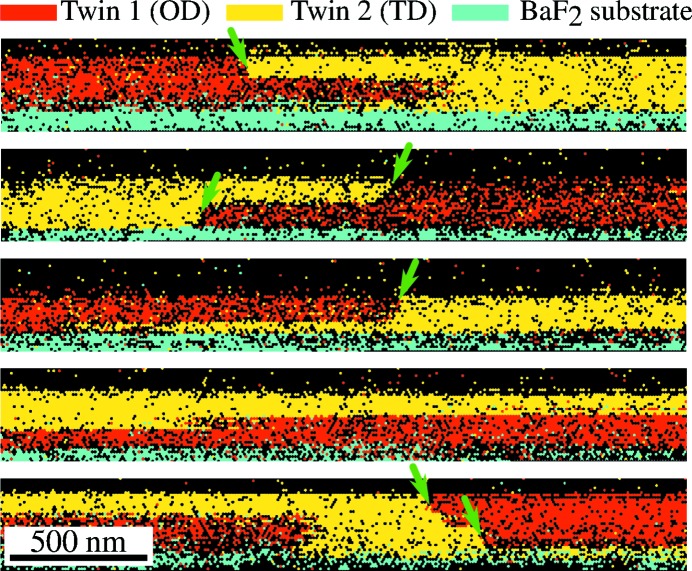
Cross-sectional EBSD images of the Bi_2_Te_3_ film BT-B. The different panels show the twin distribution at different regions along the prepared layer cross section. Yellow and red colours indicate the two twin orientations in the thin film, while cyan marks the single-crystalline BaF_2_ substrate. The shape of the interface between the two domains is found to be complicated with frequent vertical overlap of the two domains. Tilted domain boundaries are indicated by green arrows. The lower quality of these data as compared to the top view data is caused by strong charging of the insulating BaF_2_ substrate.

To shed more light on the vertical twin domain distribution over the film depth we have recorded EBSD maps on film cross sections. Fig. 7 ▸ shows such cross-sectional EBSD images of the same Bi_2_Te_3_ sample BT-B, which represent the substrate in cyan colour and the two twin orientations 1 and 2 in red and yellow, respectively. These cross-sectional images clearly reveal that the twin domain boundaries indeed have various trajectories across the film volume and that in many instances the twin domain at the surface occurs in a region with opposite twin orientation. This clearly demonstrates that the twins do not exclusively originate from the nucleation on the substrate surface, but also result from a switching of orientation during growth, *i.e.* that twinning is an intrinsic phenomenon in the structure of these van der Waals bonded materials. In Fig. 7 ▸ the resolution of the EBSD images of ∼10 nm is not really sufficient to exactly derive the orientation of the twin domain boundaries (green arrows), but the images indicate two predominant propagation directions. One is horizontal, corresponding to twinning across adjacent (000.1) planes, and the second is inclined to the surface normal by about 20–30°, which we therefore relate to the [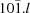] directions with *l* = 3–5. High-resolution cross-sectional TEM studies of Bi_2_Te_3_, however, suggest a more complicated trajectory of the twin boundaries not related to any crystallographic direction  (Kampmeier *et al.*, 2015 ▸).

## 4. Thickness dependence in Bi_2_Se_3_   

**Figure 8:**
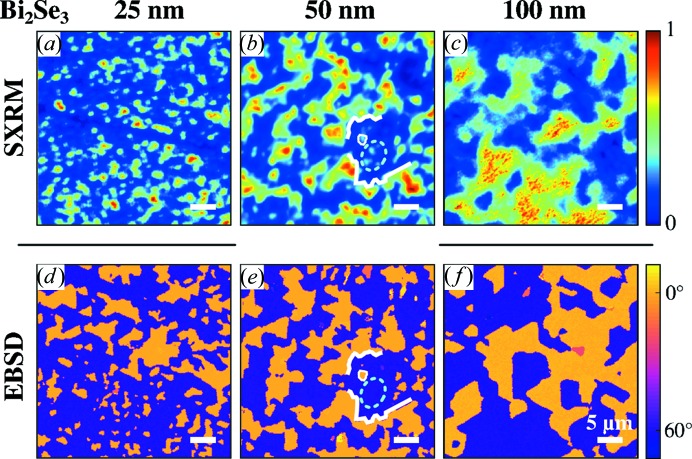
SXRM images and EBSD maps of three Bi_2_Se_3_ samples with different layer thicknesses of 25, 50 and 100 nm. The SXRM image recorded using the asymmetric (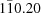) Bragg peak shown in (*a*)–(*c*) is compared with the in-plane angle of the crystal orientation determined from EBSD shown in (*d*)–(*f*). The measurements of the 50 nm-thick area were performed on the same location. A darker region/line in the SXRM image in panel (*a*) is caused by a cleavage step on the substrate surface.

To demonstrate the vast amount of information which is obtained from the application of these imaging methods, EBSD and SXRM studies of Bi_2_Se_3_ films were performed in order to evaluate the thickness evolution of the twin domain structure. Fig. 8 ▸ shows SXRM and EBSD images of three samples with increasing thicknesses of the Bi_2_Se_3_ layer of 25, 50 and 100 nm. Note that only the SXRM and EBSD images of the 50 nm-thick sample were recorded at the same sample location. As for Bi_2_Te_3_, we find a clear correspondence between the SXRM and EBSD domain images. Similar features are highlighted by white lines, while a cyan ellipse marks the differences; again, diffracted intensity is observed in the bulk-sensitive SXRM, while the opposite twin orientation is detected at the surface by EBSD.

Even though the measurements were not all performed at the same sample locations, both methods evidence a very rapid increase of the lateral twin domain size with increasing film thickness. Table 1 ▸ lists the average surface domain size determined from large-area EBSD measurements, which increases from 6.1 µm^2^ for BS-25 to 22.4 µm^2^ for BS-100. SXRM, however, also shows signals which originate from the buried domain pattern, where it is reasonable to assume that the buried domains do not change their size when overgrown further, *i.e.* that the buried domain pattern is fixed once it is nucleated. Several areas in Fig. 8 ▸(*c*) indicate the presence of a domain pattern with small-sized domains buried below larger domains near the surface, which dominate the EBSD signal. It is important to note that all three samples (BS-25, BS-50, BS-100) were grown simultaneously in the same growth run and that the different thicknesses were produced by employing a sample shutter, ruling out any differences in the growth conditions as a reason for the observed change in the domain size. In addition to the dramatic increase of the average domain size, a small change of the relative twin ratio is observed. The statistical significance of the microscopic data is, however, not sufficient to draw any quantitative conclusions.

**Figure 9:**
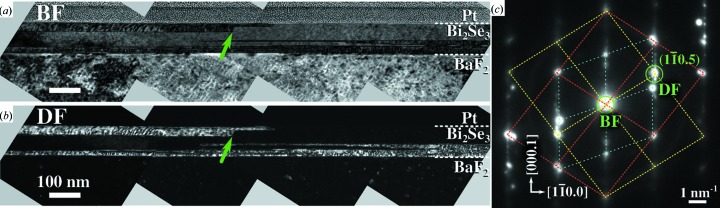
Cross-sectional bright-field (BF) and dark-field (DF) transmission electron micrographs of Bi_2_Se_3_ sample BS-100 demonstrating the twin domain distribution in the film’s cross section are shown in panels (*a*) and (*b*), respectively. The substrate/Bi_2_Se_3_ and Bi_2_Se_3_/Pt interfaces are indicated by white dashed lines. Diffraction contrast between the two twin orientations in BF imaging conditions is achieved by tilting the specimen to enhance the contribution of one domain (*c*). The DF was created using the (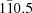) reflection where only one twin domain contributes. The limited vertical extent of a tilted twin domain is marked by a green arrow. Panel (*c*) shows the diffraction image of the imaging conditions with specimen orientation close to the [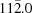] zone axis. Yellow and red colours indicate the reciprocal lattices of the two twin orientations in the thin film, while cyan marks that of the substrate.

To further clarify the twin domain distribution across the film depth, cross-sectional TEM was performed for the thickest Bi_2_Se_3_ sample BS-100. The cross section was prepared by FIB milling after the sample was covered by a protective platinum layer. The resulting TEM lamella was thinned down to a thickness of less than 100 nm for the TEM investigations. Using bright- and dark-field TEM imaging as shown in Fig. 9 ▸, the different twin domains in the electron-transparent part of the lamella were observed and a similar orientation of the defects was found as in the Bi_2_Te_3_ sample by cross-sectional EBSD. Thus, the same mechanisms for twin nucleation and formation exist in both material systems. The majority of the defect planes and domain boundaries are *c*-plane defects; however, no defect or domain fully propagates through the lamella in the image plane. The exact orientation of the lateral termination of the defects is difficult to determine since in the field of view only one termination has large enough length to allow proper observation. As for the Bi_2_Te_3_ film, the domain boundary appears to be tilted from the surface normal (*c* axis). For the given specimen thickness and resolution, the atomic structure of the defects and the domain size as a function of film depth could not be further determined.

## 5. Summary and conclusions   

In summary, we have demonstrated imaging of the twin domains and their distribution in Bi_2_Te_3_ and Bi_2_Se_3_ topological insulator thin films using EBSD and SXRM, showing the complementarity of these techniques. By investigations of the same sample areas by different techniques we revealed a clear link between the sample topography and the local crystallographic twin orientation. The borders of regions with distinct twin orientation are strictly correlated with the borders of the domains with different crystallographic orientations determined by EBSD. Surface-sensitive methods like AFM and EBSD, however, are not able to detect the twin distribution in the film depth. Using bulk-sensitive SXRM we identify the regions where one twin domain is buried below the other. Cross-sectional EBSD and TEM investigations corroborate this effect and show that the defect structure in both thin film systems consists of a combination of lateral twinning originating from the hetero-nucleation on the substrates and vertical twins arising from the switching of the orientation during the epitaxial growth process. This results in twin boundaries that regularly propagate through the film thickness at complicated trajectories. The vertically propagating defects reaching the surface are expected to influence the topological surface states. Thickness-dependent investigations further reveal a rapid growth of the average domain size with the film thickness both in the bulk and near the surface. Since the twin boundaries will cause additional carrier scattering and thus influence electrical transport properties a decreased twin defect density is important. This also explains why thicker Bi_2_Se_3_ films are more suited for topological transport studies  (Taskin *et al.*, 2012 ▸) and therefore represent a promising template towards electrical devices made from these materials.

Future experiments should focus on clarification of the exact propagation direction and structure of the vertical defects, which was not possible from our work. Advanced high-resolution electron imaging techniques can be used to study the atomic structure of the defects. Coherent diffraction imaging or ptychography will be valuable tools to improve the resolution of SXRM well below the size of the X-ray beam. By exploiting such an improved resolution it would be possible to study the propagation direction of the domain walls in all three dimensions.

## Figures and Tables

**Table 1:** Overview of studied Bi_2_ *X*_3_ samples The thin film material, film thickness, growth temperature 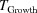 and average surface domain size determined from EBSD are listed.

Label	Material	Thickness (nm)	*T*_growth_ (K)	Average domain size (µm^2^)
BT-A	Bi_2_Te_3_	200	553	
BT-B	Bi_2_Te_3_	200	593	24.9
BS-25	Bi_2_Se_3_	25	633	6.1
BS-50	Bi_2_Se_3_	50	633	7.7
BS-100	Bi_2_Se_3_	100	633	22.4

## Footnotes

1 We use Miller indices (*hkl*) and Bravais indices (*hki.l*) with  to denote the rhombohedral structure.

## References

[bb1] Aramberri, H., Cerdá, J. I. & Muñoz, M. C. (2015). *Nano Lett.* **15**, 3840–3844. 10.1021/acs.nanolett.5b00625 25955766

[bb2] Caha, O., Dubroka, A., Humlíček, J., Holý, V., Steiner, H., Ul-Hassan, M., Sánchez-Barriga, J., Rader, O., Stanislavchuk, T. N., Sirenko, A. A., Bauer, G. & Springholz, G. (2013). *Cryst. Growth Des.* **13**, 3365–3373.

[bb3] Chahine, G. A., Richard, M.-I., Homs-Regojo, R. A., Tran-Caliste, T. N., Carbone, D., Jacques, V. L. R., Grifone, R., Boesecke, P., Katzer, J., Costina, I., Djazouli, H., Schroeder, T. & Schülli, T. U. (2014). *J. Appl. Cryst.* **47**, 762–769.

[bb4] Chahine, G. A., Zoellner, M. H., Richard, M.-I., Guha, S., Reich, C., Zaumseil, P., Capellini, G., Schroeder, T. & Schülli, T. U. (2015). *Appl. Phys. Lett.* **106**, 071902.

[bb5] Chen, Y. L., Analytis, J. G., Chu, J.-H., Liu, Z. K., Mo, S.-K., Qi, X. L., Zhang, H. J., Lu, D. H., Dai, X., Fang, Z., Zhang, S. C., Fisher, I. R., Hussain, Z. & Shen, Z.-X. (2009). *Science*, **325**, 178–181. 10.1126/science.1173034 19520912

[bb6] Guo, X., Xu, Z. J., Liu, H. C., Zhao, B., Dai, X. Q., He, H. T., Wang, J. N., Liu, H. J., Ho, W. K. & Xie, M. H. (2013). *Appl. Phys. Lett.* **102**, 151604.

[bb7] Hrauda, N., Zhang, J., Wintersberger, E., Etzelstorfer, T., Mandl, B., Stangl, J., Carbone, D., Holý, V., Jovanović, V., Biasotto, C., Nanver, L. K., Moers, J., Grützmacher, D. & Bauer, G. (2011). *Nano Lett.* **11**, 2875–2880. 10.1021/nl2013289 PMC3136111 21627099

[bb8] Kampmeier, J., Borisova, S., Plucinski, L., Luysberg, M., Mussler, G. & Grützmacher, D. (2015). *Cryst. Growth Des.* **15**, 390–394.

[bb9] Nakajima, S. (1963). *J. Phys. Chem. Solids*, **24**, 479–485.

[bb10] Richardella, A., Kandala, A., Lee, J. S. & Samarth, N. (2015). *APL Mater.* **3**, 083303.

[bb11] Richardella, A., Zhang, D. M., Lee, J. S., Koser, A., Rench, D. W., Yeats, A. L., Buckley, B. B., Awschalom, D. D. & Samarth, N. (2010). *Appl. Phys. Lett.* **97**, 262104.

[bb12] Schäfer, N., Chahine, G. A., Wilkinson, A. J., Schmid, T., Rissom, T., Schülli, T. U. & Abou-Ras, D. (2016). *J. Appl. Cryst.* **49**, 632–635.

[bb13] Schreyeck, S., Tarakina, N. V., Karczewski, G., Schumacher, C., Borzenko, T., Brüne, C., Buhmann, H., Gould, C., Brunner, K. & Molenkamp, L. W. (2013). *Appl. Phys. Lett.* **102**, 041914.

[bb14] Schwartz, A., Kumar, M., Adams, B. & Field, D. (2009). Editors. *Electron Backscatter Diffraction in Materials Science*, 2nd ed. New York: Springer.

[bb15] Stangl, J., Mocuta, C., Diaz, A., Metzger, T. H. & Bauer, G. (2009). *ChemPhysChem*, **10**, 2923–2930. 10.1002/cphc.200900563 19856372

[bb16] Tarakina, N. V., Schreyeck, S., Luysberg, M., Grauer, S., Schumacher, C., Karczewski, G., Brunner, K., Gould, C., Buhmann, H., Dunin-Borkowski, R. E. & Molenkamp, L. W. (2014). *Adv. Mater. Interfaces*, **1**, 1400134.

[bb17] Taskin, A. A., Sasaki, S., Segawa, K. & Ando, Y. (2012). *Phys. Rev. Lett.* **109**, 066803. 10.1103/PhysRevLett.109.066803 23006293

[bb18] Zhang, H., Liu, C.-X., Qi, X.-L., Dai, X., Fang, Z. & Zhang, S.-C. (2009). *Nat. Phys.* **5**, 438–442.

